# Enhanced chemokine-receptor expression, function, and signaling in healthy African American and scleroderma-patient monocytes are regulated by caveolin-1

**DOI:** 10.1186/s13069-015-0028-7

**Published:** 2015-06-20

**Authors:** Rebecca Lee, Charles Reese, Beth Perry, Jonathan Heywood, Michael Bonner, Marina Zemskova, Richard M. Silver, Stanley Hoffman, Elena Tourkina

**Affiliations:** Division of Rheumatology and Immunology, Department of Medicine, Medical University of South Carolina, 96 Jonathan Lucas Street Suite 816, MSC 637, Charleston, SC 29425 USA; Department of Regenerative Medicine and Cell Biology, Medical University of South Carolina, 96 Jonathan Lucas Street Suite 816, Charleston, SC 29425 USA

## Abstract

**Background:**

A major health disparity suffered by African Americans (AA) is a predisposition toward fibrotic diseases of the skin, lung, and other organs. We previously showed that healthy AA and scleroderma (systemic sclerosis (SSc)) patient monocytes share biochemical and functional differences from control Caucasian (C) monocytes that may predispose AA to SSc. The central difference is a decrease in caveolin-1. Low caveolin-1 levels promote monocyte migration, their differentiation into fibrocytes, and fibrocyte recruitment into fibrotic tissues. Here we have greatly expanded our studies on the mechanism of action in fibrosis of caveolin-1 in AA and SSc monocytes.

**Results:**

Expression of chemokine receptors (CCR1, CCR2, CCR3) is enhanced in healthy AA monocytes compared to healthy C monocytes and further increased in SSc monocytes. A parallel increase in function occurs assessed by migration toward chemokines MCP-1 and MCP-3. Chemokine-receptor expression and function are inhibited by the caveolin-1 scaffolding domain peptide (CSD) via its action as a surrogate for caveolin-1. Cells bearing chemokine receptors accumulate to high levels in fibrotic lung and skin tissue from SSc patients and from mice treated with bleomycin. This accumulation is almost completely blocked in mice treated with CSD. In signaling studies, Src activation is enhanced in AA monocytes compared to C monocytes and further increased in SSc monocytes. Lyn is also highly activated in SSc monocytes. Src and Lyn activation are inhibited by CSD. Src and Lyn’s roles in monocyte migration were demonstrated using specific inhibitors.

**Conclusions:**

To the best of our knowledge, this is the first report that the expression and function of CCR1, CCR2, and CCR3 are upregulated in monocytes from healthy AA and from SSc patients via molecular mechanisms involving caveolin-1, Src/Lyn, and MEK/ERK. The results suggest that the migration/recruitment of monocytes and fibrocytes into fibrotic tissues, mediated at least in part by CCR1, CCR2, and CCR3, plays a major role in the progression of lung and skin fibrosis and in the predisposition of AA to fibrotic diseases. Our findings further suggest that chemokine receptors and signaling molecules, particularly caveolin-1, that control their expression/function are promising targets for treating fibrotic diseases.

**Electronic supplementary material:**

The online version of this article (doi:10.1186/s13069-015-0028-7) contains supplementary material, which is available to authorized users.

## Background

A major health disparity affecting African Americans (AA) is a predisposition toward fibrotic diseases of the skin, lung, and other organs. AA scleroderma (systemic sclerosis, SSc) patients have a younger age of disease onset, higher probability of the more severe diffuse cutaneous form of the disease, and higher mortality. AA SSc patients are significantly more likely than Caucasian (C) SSc patients to exhibit impaired lung function [1–8]. While there has been a considerable focus on AA SSc patients, there have been few studies on underlying differences between healthy AA and C that might explain the predisposition of AA to SSc and interstitial lung disease (ILD). In one study, levels of the profibrotic cytokine transforming growth factor β (TGFβ) were twice as high in serum from healthy AA compared to healthy C [9].

We recently identified several parameters in which healthy AA are similar to SSc patients that may predispose AA to fibrosing diseases, e.g., SSc [10]. The central observation was a diminution in the master regulatory protein caveolin-1 in monocytes from healthy AA compared to healthy C. A greater loss of monocyte caveolin-1 is linked to lung and skin fibrosis in bleomycin-treated mice and in SSc-ILD and IPF patients [11–14]. The low level of caveolin-1 in AA and SSc monocytes strongly promotes their migration toward several chemokines and their differentiation into α-smooth muscle actin (ASMA)-positive fibrocytes. Both of these functions are blocked by the caveolin-1 scaffolding domain peptide (CSD), which enters cells and compensates for the lack of caveolin-1.

Monocyte migration in vitro models their recruitment in vivo into tissues undergoing inflammation and fibrosis. In both cases, chemokines provide a chemotactic signal to cells by binding to their specific cell-surface receptors. The molecular mechanism through which low caveolin-1 enhances monocyte migration involves the accumulation of chemokine receptors such as CXCR4 and CCR5 [12, 15]. This accumulation may result from either enhanced expression or decreased turnover. Signaling downstream from the chemokine receptor-ligand interaction is mediated by several pathways including G protein-coupled receptor signaling, Src-family signaling, and MAPK family signaling [16, 17]. Src-family kinases are also important in fibrosis due to their ability to regulate ECM protein expression by dermal fibroblasts.

Here we expand our study of the regulation of AA and SSc monocyte migration to additional chemokines, chemokine receptors, and signaling pathways. In particular, we have studied chemokine receptors CCR1, CCR2, and CCR3 and the chemokines MCP-1 (also known as CCL2, binds to CCR2) and MCP-3 (also known as CCL7; binds to CCR1, CCR2, and CCR3). Both MCP-1 and MCP-3 are upregulated in SSc [18]. To the best of our knowledge, this is the first report that the expression and function of CCR1, CCR2, and CCR3 are upregulated in monocytes from healthy AA and from SSc patients via molecular mechanisms involving caveolin-1, Src/Lyn, and MEK/ERK signaling.

## Results

### CCR1, CCR2, and CCR3 expression and function are enhanced in monocytes from healthy AA and SSc patients

**Fig. 1:**
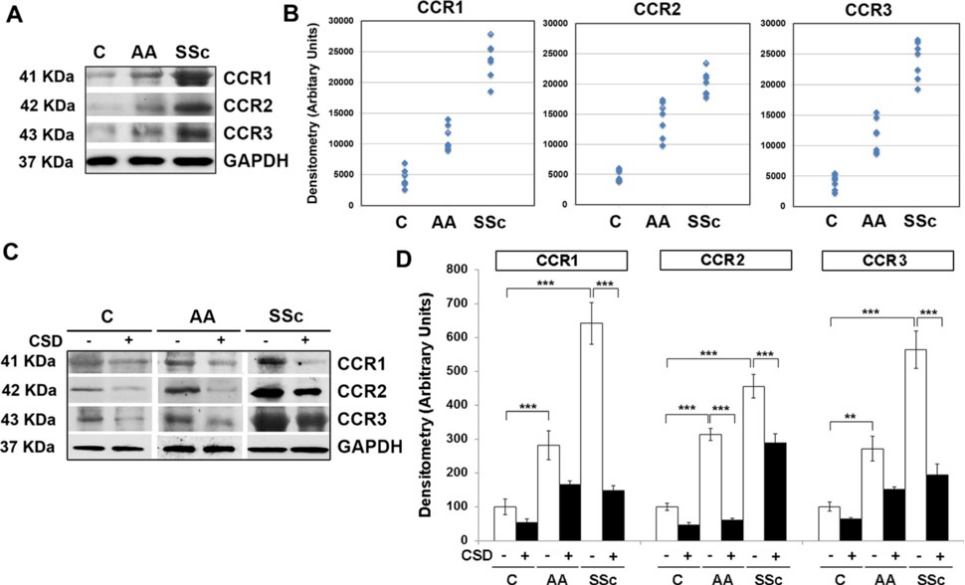
Enhanced CCR1, CCR2, and CCR3 expression in AA and SSc monocytes. **a** Extracts of monocytes from the indicated subjects (50 μg total protein per lane) from a representative experiment were Western blotted using the indicated primary antibodies. GAPDH was used as the loading control. **b** Densitometric quantification of CCR1, CCR2, and CCR3 levels in monocytes from the indicated subjects. Values for individual subjects are shown (*n* = 7). **c** Western blot of a representative experiment using the indicated primary antibodies in which monocytes were treated with CSD (+) or control peptide (−). GAPDH was used as the loading control. **d** The average level of CCR1, CCR2, or CCR3 in C monocytes treated with control peptide was set to 100 arbitrary units. *Bars* show the mean ± SEM from four independent experiments performed with cells from different subjects. ***p < 0.002; **p < 0.01; *p < 0.05

**Fig. 2:**
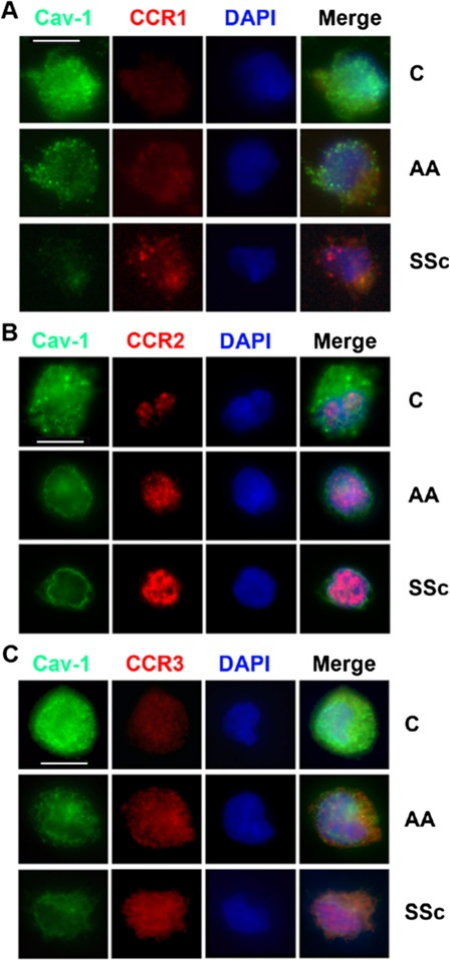
Demonstration by immunocytochemistry of increased expression of CCR1, CCR2, and CCR3 and decreased expression of caveolin-1 by AA and SSc monocytes. C, AA, and SSc monocytes were stained *green* for caveolin-1 (Cav-1) and *red* for CCR1 (**a**), CCR2 (**b**), or CCR3 (**c**) as described in the “Methods” section. Nuclei were counterstained using DAPI (*blue*). Representative images are shown typical of the results obtained in four independent experiments in each category. *Bars* = 5 μm

We reported that expression of the chemokine receptor CXCR4 is enhanced in healthy AA monocytes compared to healthy C monocytes and that it is present at a still higher level in SSc patient monocytes [10]. Similarly, we showed enhanced CXCR4 function in that we observed enhanced migration of healthy AA and SSc patient monocytes toward the CXCR4 ligand SDF-1. To expand on these studies, here we examine additional chemokine receptors: CCR1, CCR2, and CCR3. Western blot analyses reveal increases in the expression of all three receptors in AA and SSc monocytes (Fig. 1). As with CXCR4, treatment of AA and SSc monocytes with CSD decreases the expression of these chemokine receptors down to the level observed in C monocytes (Fig. 1c, d). The results of these Western blot experiments were validated in IHC experiments that demonstrated decreased levels of caveolin-1 and increased levels of CCR1, CCR2, and CCR3 in AA and SSc monocytes compared to C monocytes (Fig. 2).

**Fig. 3:**
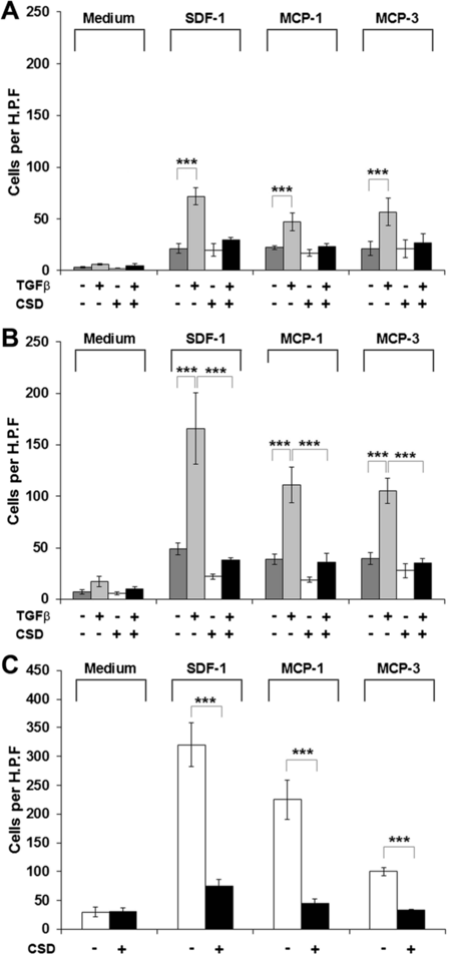
Migration toward various chemokines. Migration experiments were performed as described in the “Methods” section using C (**a**), AA (**b**), or SSc (**c**) monocytes. Cells were treated with TGFβ and CSD as indicated. Chemokines used as chemoattractants (and medium only control) are indicated. Data are expressed in terms of number of migrating cells counted per high power field. *Bars* show the mean level of migration ± SEM from four different subjects in each category determined in independent experiments. ***p < 0.002; **p < 0.01; *p < 0.05

To determine whether chemokine receptor function is also enhanced in healthy AA and SSc patient monocytes, we evaluated monocyte migration toward chemokines MCP-1, MCP-3, and SDF-1. Almost no migration occurred in the absence of chemokines (Fig. 3, “Medium”). For each chemokine, the basal rate of migration was higher for healthy AA monocytes (Fig. 3b) than for healthy C monocytes (Fig. 3a) and higher still for SSc monocytes (Fig. 3c), especially for SDF-1 and MCP-1. When migration was examined in cells activated with TGFβ, again migration toward each chemokine was higher for healthy AA monocytes than for healthy C monocytes. In all cases, migration was strongly inhibited when cells were treated with CSD.

### CCR1, CCR2, and CCR3 are upregulated in SSc patient lung and skin tissue

**Fig. 4:**
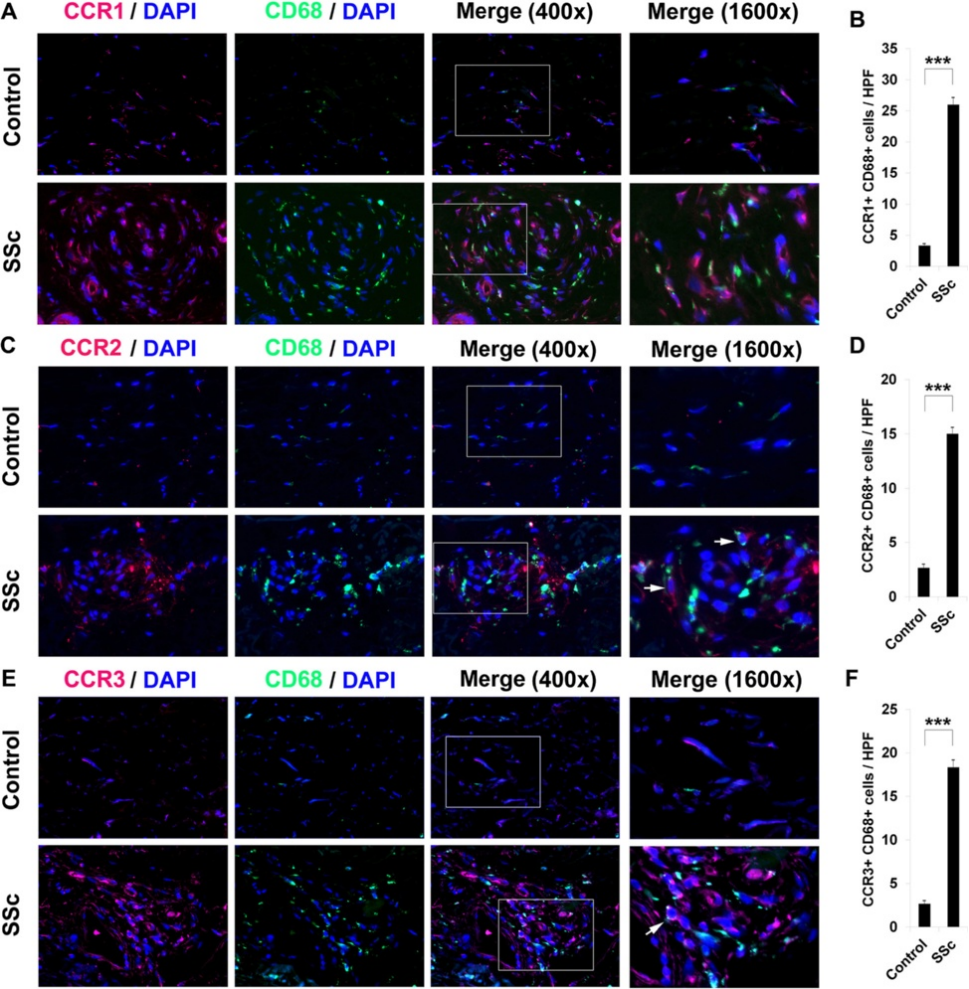
Overexpression of CCR1, CCR2, CCR3, and CD68 in scleroderma skin tissue. Control and SSc skin sections were double-stained for the monocyte/macrophage marker CD68 (*green*) and for **a** CCR1, **c** CCR2, or **e** CCR3 (*red*). Nuclei were counterstained using DAPI (*blue*). The indicated fields in the third column are shown at high magnification in the fourth column. Note the enhanced expression of all of these proteins in SSc skin compared to Control skin. Note (see *arrows*) that the green and red staining in double-labeled cells need not overlap. Similar results were obtained in three independent samples in each category. **b**, **d**, **f** Quantification of staining. Representative fields were photographed at 400× magnification. The number of double-positive cells (**b**, CCR1+/HSP47+; **d**, CCR2+/HSP47+; **f**, CCR3+/HSP47+) was counted in three subjects per category, five high power fields (HPF) per subject. The data are presented in terms of the number of double-positive cells per HPF (average ± SEM). ***p < 0.002; **p < 0.01; *p < 0.05

**Fig. 5:**
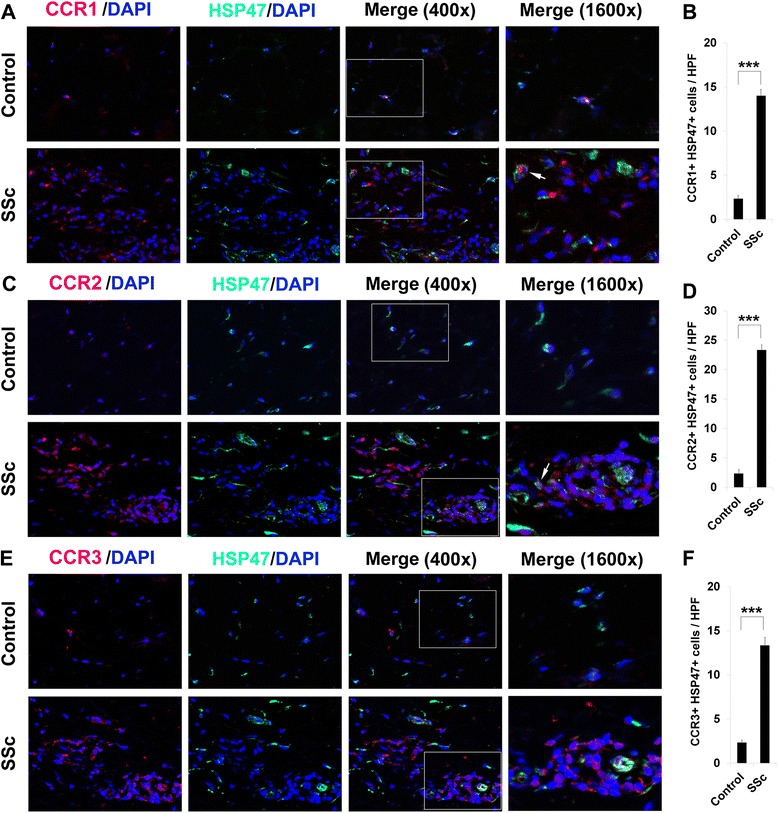
Overexpression of CCR1, CCR2, CCR3, and HSP47 in scleroderma skin tissue. Control and SSc skin sections were double-stained for the collagen chaperone HSP47 (*green*) and for **a** CCR1, **c** CCR2, or **e** CCR3 (*red*). Nuclei were counterstained using DAPI (*blue*). The indicated fields in the third column are shown at high magnification in the fourth column. Note the enhanced expression of all of these proteins in SSc skin compared to Control skin. Note (see *arrows*) that the green and red staining in double-labeled cells need not overlap. Similar results were obtained in three independent samples in each category. **b**, **d**, **f** Quantification of staining. Representative fields were photographed at 400× magnification. The number of double-positive cells (**b**, CCR1+/HSP47+; **d**, CCR2+/HSP47+; **f**, CCR3+/HSP47+) was counted in three subjects per category, five high power fields (HPF) per subject. The data are presented in terms of the number of double-positive cells per HPF (average ± SEM). ***p < 0.002; **p < 0.01; *p < 0.05

**Fig. 6:**
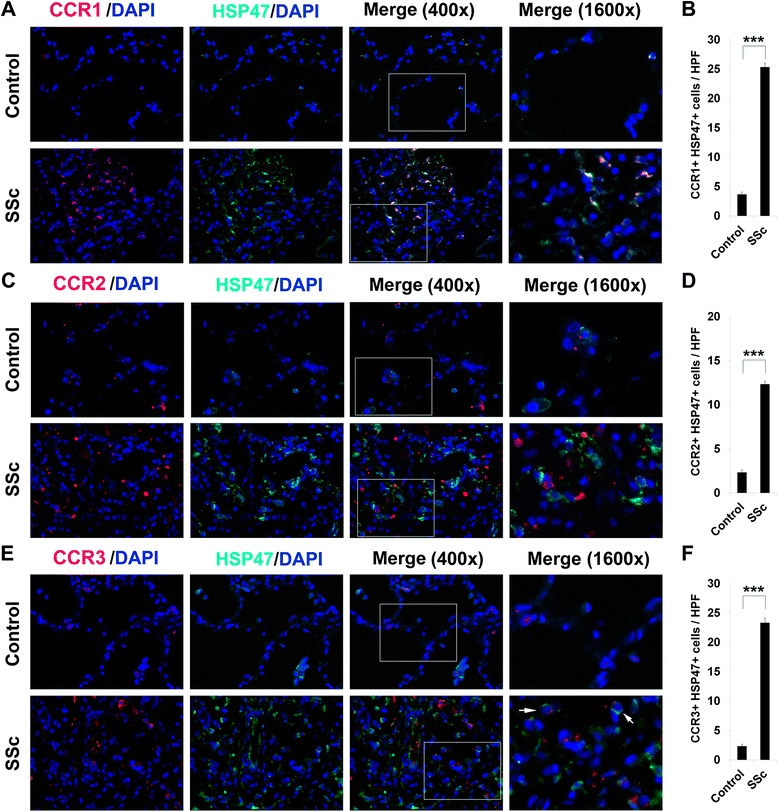
Overexpression of CCR1, CCR2, and CCR3 and HSP47 in scleroderma lung tissue. Control and SSc lung sections were double-stained for HSP47 (*green*) and for **a** CCR1, **c** CCR2, or **e** CCR3 (*red*). Nuclei were counterstained using DAPI (*blue*). The indicated fields in the third column are shown at high magnification in the fourth column. Note the enhanced expression of all of these proteins in SSc lung compared to Control lung. Note (see *arrows*) that the green and red staining in double-labeled cells need not overlap. Similar results were obtained in three independent samples in each category. **b**, **d**, **f** Quantification of staining. Representative fields were photographed at 400× magnification. The number of double-positive cells (**b**, CCR1+/HSP47+; **d**, CCR2+/HSP47+; **f**, CCR3+/HSP47+) was counted in three subjects per category, five high power fields (HPF) per subject. The data are presented in terms of the number of double-positive cells per HPF (average ± SEM). ***p < 0.002; **p < 0.01; *p < 0.05

Given that CCR1, CCR2, and CCR3 are upregulated on SSc monocytes, we also compared their expression in SSc and control lung and skin tissue. To begin to determine which cell types express these chemokine receptors in SSc, these studies were performed as double-label experiments with the monocyte/macrophage marker CD68 or with the collagen chaperone HSP47 (which serves as a marker for fibrocytes and fibroblasts). Little staining was observed in control tissue, except occasionally in alveolar macrophages; however, prevalent double staining was observed in SSc skin (Fig. 4, CD68 and chemokine receptors; Fig. 5, HSP47 and chemokine receptors) and lung tissue (Fig. 6, HSP47 and chemokine receptors). It may be noteworthy that among CCR1, CCR2, and CCR3/HSP47 double staining, CCR2 double staining was the most prominent in SSc skin (Fig. 5d) and the least prominent in SSc lung (Fig. 6d). In contrast, CCR2/ CD68 double staining was the least prominent in SSc (Fig. 4d).

### Upregulation of CCR1, CCR2, and CCR3 in fibrotic mouse tissues is reversed by CSD

**Fig. 7:**
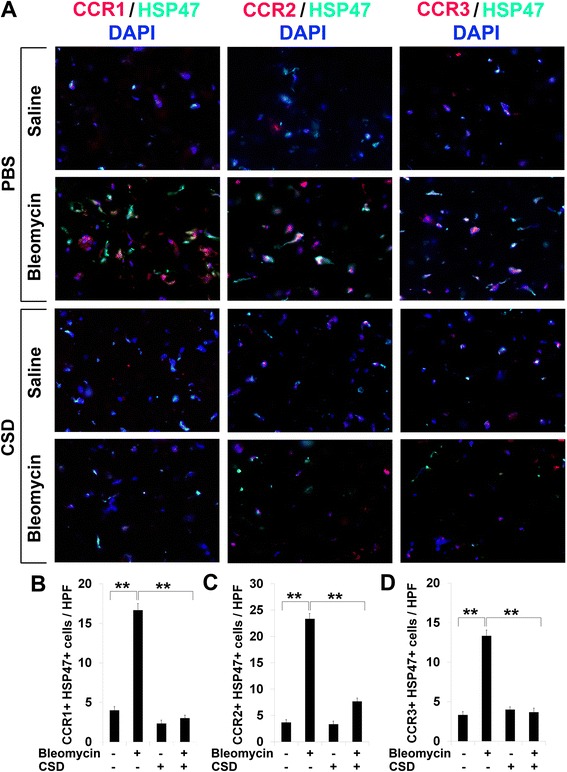
Overexpression of CCR1, CCR2, and CCR3 in fibrotic mouse skin is reversed by CSD. **a** Skin tissue sections from mice treated systemically with bleomycin or vehicle and injected daily i.p. with CSD or vehicle were double-stained for CCR1, CCR2, or CCR3 (*red*) and for HSP47 (*green*). Nuclei were counterstained using DAPI (*blue*). Note the enhanced expression of all of these proteins in the skin tissue of bleomycin-treated mice and that CSD treatment almost completely blocked the accumulation of these proteins. Similar results were obtained in three independent mice in each category. **b**–**d** Quantification of staining. Representative fields were photographed at 400× magnification. The number of double-positive cells (**b**, CCR1+/HSP47+; **c**, CCR2+/HSP47+; **d**, CCR3+/HSP47+) was counted in three subjects per category, five high power fields (HPF) per subject. The data are presented in terms of the number of double-positive cells per HPF (average ± SEM). ***p < 0.002; **p < 0.01; *p < 0.05

**Fig. 8:**
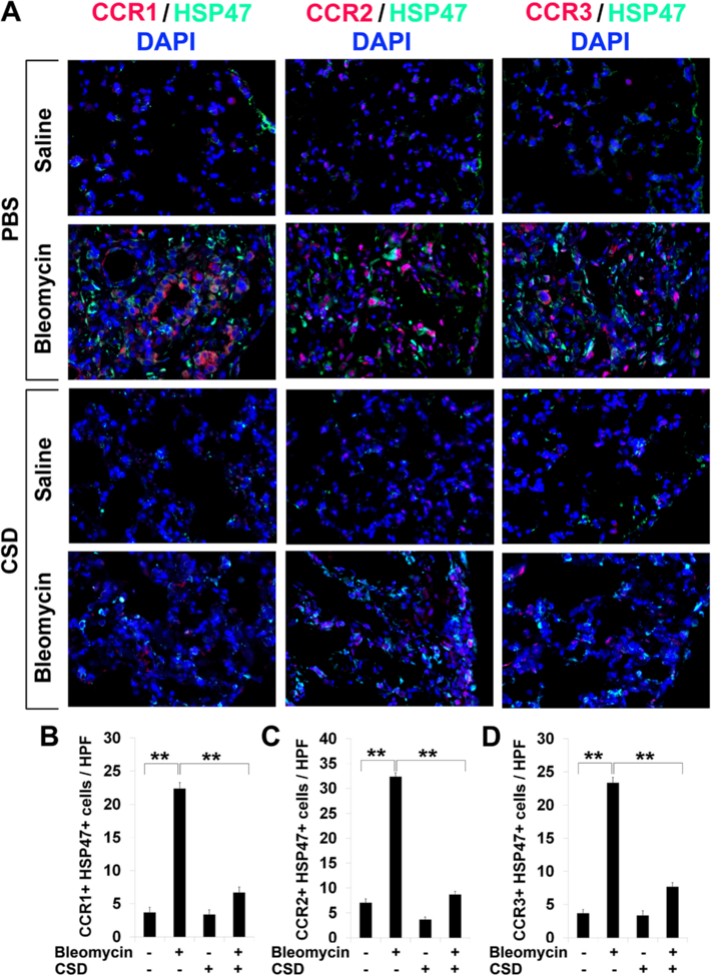
Overexpression of CCR1, CCR2, and CCR3 in fibrotic mouse lung tissue is reversed by CSD. **a** Lung tissue sections from mice treated systemically with bleomycin or vehicle and injected daily i.p. with CSD or vehicle were stained for CCR1, CCR2, or CCR3 (*red*) and for HSP47 (*green*). Nuclei were counterstained using DAPI (*blue*). Note the enhanced expression of all of these proteins in the lung tissue of bleomycin-treated mice and that CSD treatment almost completely blocked the accumulation of these proteins. Similar results were obtained in three independent mice in each category. **b**–**d** Quantification of staining. Representative fields were photographed at 400× magnification. The number of double-positive cells (**b**, CCR1+/HSP47+; **c**, CCR2+/HSP47+; **d**, CCR3+/HSP47+) was counted in three subjects per category, five high power fields (HPF) per subject. The data are presented in terms of the number of double-positive cells per HPF (average ± SEM). ***p < 0.002; **p < 0.01; *p < 0.05

We recently used a mouse model system in which bleomycin is delivered systemically by subcutaneously implanted osmotic minipumps (Pump Model) to induce fibrosis in multiple organs including lungs and skin [19], and to demonstrate that the development of fibrosis in both lungs and skin is blocked by treatment with CSD [15, 20]. Here we examine the expression of CCR1, CCR2, and CCR3 in this murine bleomycin pump model. As in SSc patients, double staining for these molecules and HSP47 in fibrotic skin (Fig. 7) and lung (Fig. 8) tissue was greatly enhanced compared to control tissue. Treatment with CSD brought the level of expression of CCR1, CCR2, and CCR3 down essentially to the level observed in control (saline-treated) animals (Figs. 7 and 8). These observations support the idea that CSD blocks the progression of fibrosis by inhibiting the recruitment/accumulation in target tissues of cells expressing CCR1, CCR2, and/or CCR3.

### Enhanced Src and Lyn activation in AA and SSc monocytes

**Fig. 9:**
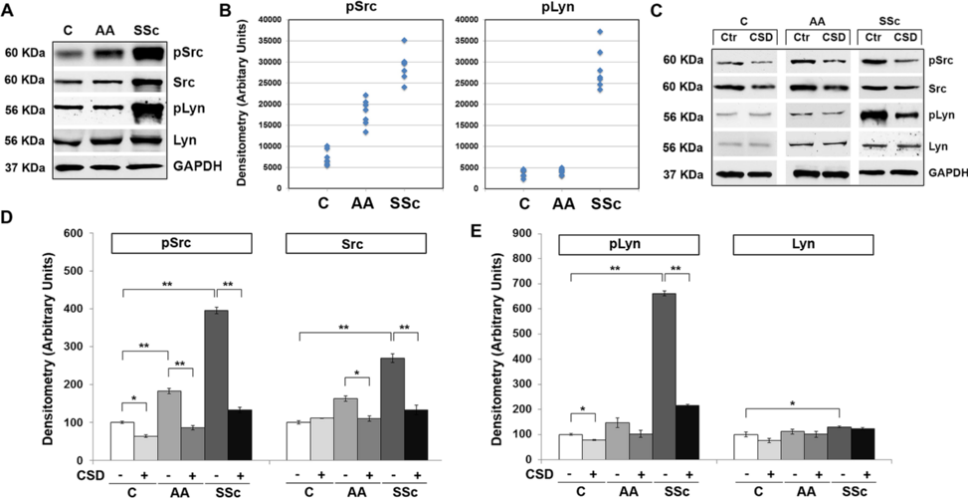
Enhanced Src and Lyn expression/activation in AA and SSc monocytes. **a** Extracts of monocytes from the indicated subjects (50 μg total protein per lane) from a representative experiment were analyzed by Western blot using the indicated primary antibodies. GAPDH was used as the loading control. **b** Densitometric quantification of pSrc and pLyn levels in monocytes from the indicated subjects. Values for individual subjects are shown (*n* = 7). **c** Western blot of a representative experiment using the indicated primary antibodies in which monocytes were treated with CSD (+) or control peptide (−). GAPDH was used as the loading control. **d**, **e** The average level of pSrc, Src, pLyn, and Lyn in C monocytes treated with control peptide was set to 100 arbitrary units. Note that both Src and Lyn are highly activated in SSc monocytes while only Src is activated in AA monocytes. *Bars* show the mean ± SEM from four independent experiments performed with cells from different subjects. ***p < 0.002; **p < 0.01; *p < 0.05

To identify signaling mechanisms responsible for the hypermigration of SSc and AA monocytes, we studied kinases activated by chemokine-receptor interactions. We demonstrated that ERK is activated in AA and SSc monocytes [10, 12]. Here we show that both Src and Lyn are highly activated in SSc monocytes, and that Src, but not Lyn, is also activated in AA monocytes (Fig. 9a, b). CSD treatment of monocytes inhibited Src and Lyn activation in SSc monocytes and Src activation in AA monocytes (Fig. 9c–e). Thus, Src and Lyn may be critical players in the altered behavior of SSc and AA monocytes.

### MEK/ERK and Src-family inhibitors inhibit monocyte migration

**Fig. 10:**
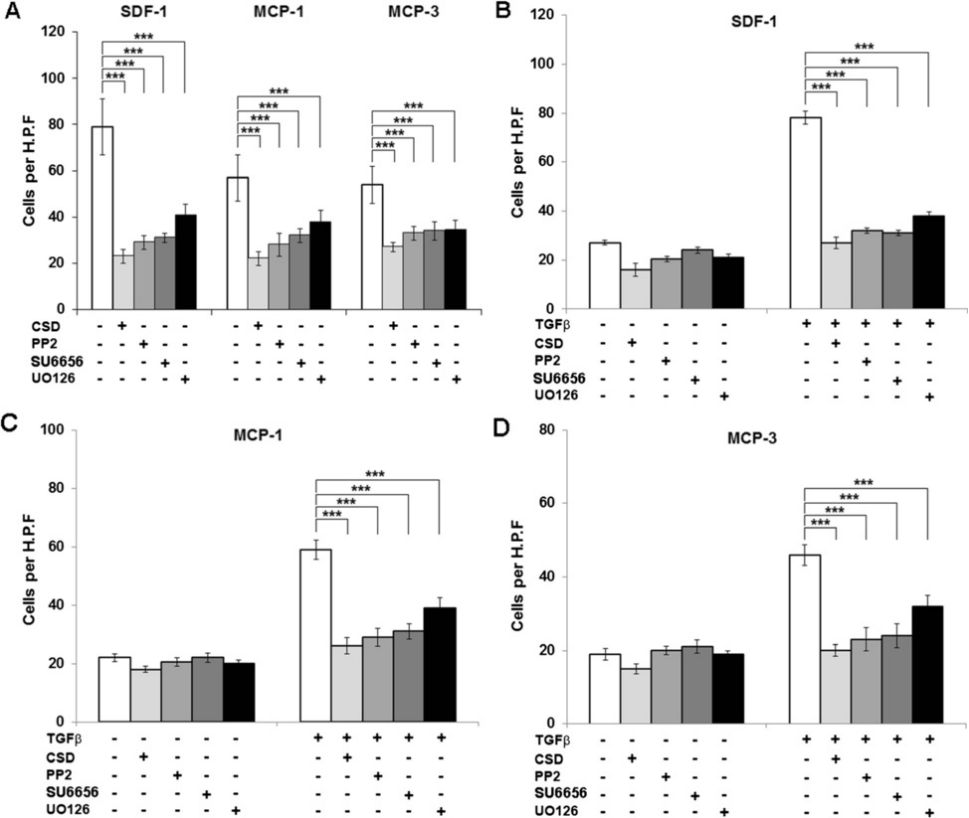
MEK/ERK and Lyn/Src kinase inhibitors inhibit monocyte migration toward SDF-1, MCP-1, and MCP-3. Migration experiments were performed as described in the Methods using SSc (**a**) or C (**b–d**) monocytes. Chemokines used are indicated as are the treatment of monocytes with TGFβ, CSD, PP2, SU6656, and U0126. Data are expressed in terms of number of migrating cells counted per high power field. *Bars* show the mean level of migration ± SEM from four different subjects in each category determined in independent experiments. Note that while PP2, SU6656, and U0126 inhibit the migration of SSc monocytes and of C monocytes activated using TGFβ, they are not as effective as CSD. ***p < 0.002; **p < 0.01; *p < 0.05

To validate the functional importance of MEK/ERK and Src/Lyn in monocyte migration, we used the MEK/ERK inhibitor U0126 and Src/Lyn inhibitors PP2 and SU6656. All these reagents significantly inhibited SSc monocyte migration toward SDF-1, MCP-1 and MCP-3, although not as effectively as CSD (Fig. 10a). Similarly, all these reagents significantly inhibited the migration of TGFβ-activated C monocytes, although again, not as effectively as CSD (Fig. 10b–d). Neither CSD nor these reagents significantly inhibited the low level of basal migration observed in control C monocytes not activated with TGFβ. While CSD did significantly inhibit the migration of control AA monocytes (not treated with TGFβ) [10], U0126, PP2, and SU6656 did not significantly inhibit their migration (data not shown). It is likely that CSD is more effective than U0126, PP2, or SU6656 in inhibiting migration because CSD inhibits multiple signaling cascades while each of these reagents inhibits only a single cascade.

## Discussion

In previous studies, we showed that caveolin-1 levels are low in monocytes from SSc patients and from healthy AA compared to healthy C [10, 12]. Profibrotic features associated with monocytes from both SSc-ILD and healthy AA subjects include the enhanced expression of chemokine receptors CXCR4 and CCR5, enhanced migration toward their respective chemokine ligands (SDF-1 for CXCR4, MIP-1α and MIP-1β for CCR5), and enhanced differentiation into fibrocytes [10, 12, 19]. All of these features were linked to low caveolin-1 expression by the fact that they were reversed by restoring caveolin-1 function with the CSD. Here we greatly expand on these studies, demonstrating: (1) the enhanced expression of chemokine receptors CCR1, CCR2, and CCR3 by AA and SSc-ILD monocytes and the reversal of the enhanced expression by restoring caveolin-1 function with CSD; (2) the enhanced migration of AA and SSc-ILD monocytes toward chemokines MCP-1 and MCP-3 and the reversal of the enhanced migration by CSD; (3) enhanced Lyn/Src signaling in AA and SSc-ILD monocytes, its reversal by CSD, and the use of specific inhibitors to demonstrate the regulation of monocyte migration by Lyn/Src and MEK/ERK; (4) the overexpression of CCR1, CCR2, and CCR3 in SSc skin and lung tissue; and (5) the overexpression of CCR1, CCR2, and CCR3 in fibrotic murine skin and lung tissue generated by systemic bleomycin delivery using implanted osmotic minipumps, and the reversal of this overexpression by treatment with CSD.

CCR1, CCR2, and CCR3 are expressed on a variety of classes of leukocytes. Here we have focused on their expression by monocytes. To the best of our knowledge, there have been few previous reports related to their enhanced expression by monocytes from SSc patients and healthy AA subjects. At the mRNA level, CCR1 expression was enhanced in monocytes from SSc patients with PAH [21, 22]. No data was presented at the protein level. CCR2 expression detected by immunohistochemistry was observed to be upregulated in early-stage diffuse cutaneous SSc skin by a variety of cell types including macrophages, myofibroblasts, pericytes, lymphocytes, and endothelial cells [23]. We also find a major upregulation of CCR2 in SSc skin in the fibrocyte-fibroblast lineage (HSP47+ cells). Interestingly, the number of double-positive cells in this lineage in the lungs is greater for CCR1 and CCR3 than for CCR2. Overall, comparing double staining with the monocyte/macrophage marker CD68 to double staining with HSP47 suggests that the various cell types that express chemokine receptors accumulate differentially during fibrosis.

Although key receptors can be differentially expressed on human and murine cells [24, 25], we find that CCR1, CCR2, and CCR3 are all expressed at high levels in both human and mouse fibrotic skin and lung tissue. Moreover, we find that the overexpression of these receptors is inhibited when mice are treated with CSD. Previous studies in mouse model systems are also consistent with the importance of these chemokine receptors in fibrosing disease. For example, antibodies against CCR1 delivered i.v. enhanced the survival of mice treated with bleomycin while inhibiting the accumulation in their lungs of collagen and inflammatory cells [26]. Experiments using CCR2 knockout mice demonstrated that CCR2 plays a major role in the recruitment of fibrocytes into the airspace of mice in which fibrosis was induced using FITC [27]. In yet another model, the overproduction of collagen induced by injection of TGFβ into the skin was significantly reduced in CCR2 knockout mice [28].

Chemokines MCP-1 and MCP-3 are present at high levels in the serum and bronchoalveolar lavage fluid of SSc patients and are expressed at high levels by SSc fibroblasts [23, 29–32]. Among SSc patients, high levels of MCP-1 and MCP-3 are associated with worse clinical outcomes. In addition to their role as chemoattractants of inflammatory cells into target tissues, MCP-1 and MCP-3 may be important in fibrosis as initiators of signaling cascades resulting in collagen overexpression [18, 30, 33, 34]. While some studies show direct effects of MCP-1 and MCP-3 on collagen expression by fibroblasts [30, 34, 35], another study proposes that MCP-1 indirectly promotes the expression of collagen by fibroblasts by activating the expression of IL-4 by T cells [33]. In turn, this IL-4 is responsible for increasing collagen production by fibroblasts.

Relatively little is known about the signaling pathways that link relative caveolin-1 deficiency in SSc and AA monocytes to the enhanced ability of these cells to migrate toward various chemokines and to differentiate into fibrocytes. We reported the importance of MEK/ERK signaling [10, 12] in these cell functions. Here we have studied the Src-family kinases Src and Lyn. We find that Src and Lyn are hyperactivated in SSc monocytes and that Src is activated in AA monocytes. In both cases, Src and Lyn activation are reversed by treating cells with CSD. In addition, we find that the Src/Lyn inhibitors PP2 and SU6656 (as well as a MEK/ERK inhibitor U0126) block the enhanced migration of SSc and AA monocytes. Not surprisingly, CSD (which blocks multiple signaling pathways) was slightly more effective than these inhibitors that block only one pathway. Src-family kinases have been implicated in a variety of activities relevant to monocyte biology including innate immune signaling, responses to cytokines and growth factors, apoptosis, and G protein-coupled signaling [36]. To the best of our knowledge, the current study is the first to link caveolin-1, Src-family signaling, and monocyte migration.

Caveolin-1 and Src have been studied more extensively in other cell types in the context of SSc and fibrosis. Most of these studies involve signaling initiated by TGFβ. In one study focusing on the role of urokinase-type plasminogen activator (uPA) and plasminogen activator inhibitor (PAI) in regulating the epithelial-mesenchymal transformation (EMT) of alveolar type II epithelial cells into myofibroblasts, it was proposed that CSD blocked EMT by inhibiting Src leading to the enhanced expression of uPA and the inhibition of PAI expression [37]. These effects were observed whether EMT was induced by bleomycin, TGFβ, or cigarette smoke. Other studies focus on fibroblasts. TGFβ receptor internalized through caveolin-1 lipid rafts undergoes rapid degradation, thereby decreasing TGFβ signaling [38]. This mechanism links low caveolin-1 to enhanced TGFβ signaling. It is noteworthy that TGFβ is present at high levels in the circulation of healthy AA [9] and SSc patients [39], and TGFβ treatment decreases caveolin-1 levels in a variety of cell types [12, 38]. Thus, the combination of low caveolin-1 and high TGFβ may be particularly likely to cause fibrosis because their effects appear to be mutually reinforcing. Src has also been directly linked to TGFβ signaling. Stimulation of human dermal fibroblasts with TGFβ activated Src signaling [40]. Treatment of these cells with SU6656 inhibited collagen expression both at the mRNA and protein levels. Similarly, dermal fibrosis induced in mice by bleomycin injection was inhibited by SU6656. Finally, it was observed that TGFβ can signal through the Src family member c-Abl and that this signaling is independent of canonical TGFβ signaling through Smad2/3 [41].

## Conclusions

In summary, the current study strongly supports and extends our observations on the role of monocytes and cells derived from monocytes (e.g., fibrocytes) in lung and skin fibrosis and on the predisposition of AA to fibrotic diseases. Our findings highlight the idea that chemokine receptors (e.g., CCR1, CCR2, CCR3) and signaling molecules that control their expression/function (e.g., caveolin-1, MEK/ERK, Src/Lyn) are promising targets for novel treatments for fibrotic diseases such as SSc.

## Methods

### Blood donors

Under a protocol approved by the Medical University of South Carolina (MUSC) Institutional Review Board for Human Research, SSc-ILD patients were recruited from the MUSC Scleroderma Clinic. All patients provided written informed consent before enrollment in the study, fulfilled the American College of Rheumatology criteria for SSc [42], and had evidence of ILD [12]. Demographic data for SSc patients and healthy control donors are summarized in Additional file 1: Tables S1 and S2. Note that Additional file 1: Table S1 describes the combined data for all the patients that participated in the entire study, not the patients that participated in a particular experiment.

### PBMC and monocyte isolation

Peripheral blood mononuclear cells (PBMC) were isolated by standard methods [12] by centrifugation on density 1.083 Histopaque cushions. Monocytes were isolated from the PBMC by immunodepletion using a Dynal Monocyte Negative Isolation Kit (Invitrogen, Carlsbad, CA) resulting in a cell population about 95 % Mac-1+ monocytes [12].

### Peptide treatments

The CSD peptide (amino acids 82–101 of caveolin-1; DGIWKASFTTFTVTKYWFYR) was synthesized as a fusion peptide to the C terminus of the Antennapedia Internalization Sequence (RQIKIWFQNRRMKWKK). The Antennapedia Internalization Sequence (AP) alone was used as control peptide and showed no effect on cell behavior when compared to no added peptide. When treating cells with peptides, stock solutions of peptides (10 mM in 100 % DMSO) were diluted to the indicated final concentrations.

### Monocyte migration assays

Were performed as described [11]. Briefly, SDF-1 (100 ng/ml in RPMI 1640/1 % BSA), MCP-1 or MCP-3 (50 ng/ml in RPMI 1640/1 % BSA), or unsupplemented RPMI 1640/1 % BSA were placed into the lower wells of Neuro Probe Multiwell Chemotaxis Chambers (Neuro Probe, Gaithersburg, MD) fitted with 5-μm pore size polycarbonate filters. With or without TGFβ pretreatment (45 min, 10 ng/ml in RPMI 1640/1 % BSA), 25 μl of cell suspension (5 × 10^5^ cells/ml) was placed in the upper wells. Peptides (0.1 μM) or inhibitors (U0126, 0.1 μM; PP2, 10 μM; SU6656, 10 μM) were added to the cell suspension prior to placement in the upper chamber. After incubation (2.5 h, 37 ° C, 5 % CO_2_), filters were removed, fixed, and stained with 4′,6-diamidino-2-phenylindole (DAPI) (Invitrogen, Carlsbad, CA). Cells on the underside of the membrane were photographed and counted in six high power fields per condition.

### Monocyte signaling/Western blots

Chemokine-receptor levels and levels of total and activated Src and Lyn were determined by Western blot of sodium dodecyl sulfate-polyacrylamide gel electrophoresis (SDS-PAGE) sample buffer extracts of freshly isolated monocytes. For CSD treatment, monocytes were cultured overnight in 6-well tissue culture plates (2 × 10^6^ cells per well) in RPMI 1640/20 % fetal calf serum (FCS). Attached cells were then treated for 3 h with fresh medium (RPMI/1 % BSA) supplemented with 0.1 μM CSD or control peptide. Cells were next washed twice with PBS then extracted with SDS-PAGE sample buffer. Western blots were performed using the indicated antibodies.

### Immunocytochemistry

Images were collected using a Leica DMI 4000B fluorescence microscope. To detect caveolin-1, CCR1, CCR2, and CCR3, cells isolated as described above were cultured overnight in 6-well tissue culture plates (1 × 10^6^ cells per well) on coverslips in RPMI 1640/20 % FCS. Cells were then fixed and permeabilized, labeled with appropriate primary and secondary antibodies, and counterstained with the nuclear stain DAPI.

Immunohistochemistry of human lung tissue sections was performed as described [11]. Briefly, paraffin sections were stained with primary antibodies, appropriate AlexaFluor647- or AlexaFluor555-conjugated secondary antibodies and the nuclear stain DAPI (Invitrogen, Carlsbad, CA). Images were collected using a Leica DMI 4000B fluorescence microscope. Primary antibodies were: rabbit anti-CCR1 (Thermo Fisher Scientific, Rockford, IL, USA; PA1-21629), rabbit anti-CCR2 (Abcam, Cambridge, MA, USA; ab32144), rabbit anti-CCR3 (Abcam, Cambridge, MA, USA; ab36827), rabbit anti-MCP-1 (Abcam, Cambridge, MA, USA; ab 9669), rabbit anti-MCP-3 (Santa Cruz Biotechnology, Santa Cruz, CA, USA; SC-374002), rabbit anti-pSrc-Tyr416 (Cell Signaling Technology, Inc., Danvers, MA, USA; #2101S), and rabbit anti-pLyn-Tyr507 (Cell Signaling Technology, Inc., Danvers, MA, USA; 04–375).

### Mouse experiments

Mice were treated systemically with bleomycin or vehicle and received CSD or vehicle as recently described [15, 19]. These studies were performed under protocols approved by the MUSC Institutional Animal Care and Use Committee (AR#3134, AR#3029, AR#3323).

### Statistical analyses

Immunoreactive bands were quantified by densitometry using Image J 1.32 NIH software. Raw densitometric data were processed and analyzed using Prism 3.0 (GraphPad Software Inc.). ANOVA with post hoc Tukey’s test was used to evaluate Western blots and monocyte migration. In all figures, ***indicates *p* < 0.002, **indicates *p* < 0.01, and *indicates *p* < 0.05.

## Additional file

Additional file 1:
**Summary of human subject demographics and Table S1 legend.** **Table S1.** Clinical features of SSc patients. **Table S2.** AA and Caucasian controls.

## Abbreviations

AA: African American
ANA: antinuclear antibodies
ANOVA: analysis of variance
AP: Antennapedia internalization sequence
ASMA: α-smooth muscle actin
BSA: bovine serum albumin
C: Caucasian
CCL2: 7, Chemokine (C-C motif) ligand 2, 7
CCR1: 2, 3, 5, C-C chemokine receptor types 1, 2, 3, 5
CSD: caveolin-1 scaffolding domain peptide
CXCR4: C-X-C receptor type 4
DAPI: 4′,6-diamidino-2-phenylindole
DMSO: dimethylsulfoxide
ECM: extracellular matrix
EMT: epithelial-mesenchymal transformation
ERK: extracellular signal-regulated kinase
FCS: fetal calf serum
FITC: fluorescein isothiocyanate
FVC: forced vital capacity
IL-4: interleukin-4
ILD: interstitial lung disease
IPF: idiopathic pulmonary fibrosis
MAPK: mitogen-activated protein kinase
MCP-1: MCP-3, monocyte chemotactic protein-1, monocyte chemotactic protein-3
MEK: MAPK/ERK kinase
MIP-1α: β, macrophage inflammatory protein-1α, β
MUSC: Medical University of South Carolina
PAH: pulmonary arterial hypertension
PAI: plasminogen activator inhibitor
PBMC: peripheral blood mononuclear cells
PBS: phosphate buffered saline
SDF-1: stromal derived factor-1, also known as CXCL12
SDS-PAGE: sodium dodecyl sulfate-polyacrylamide gel electrophoresis
SEM: standard error of the mean
SSc: scleroderma (systemic sclerosis)
TGFβ: transforming growth factor β
uPA: urokinase-type plasminogen activator
