# Supplementary material for: Enhanced chemokine-receptor expression, function, and signaling in healthy African American and scleroderma-patient monocytes are regulated by caveolin-1
Source: Fibrogenesis Tissue Repair. 2015 Jun 20;8:11. doi: 10.1186/s13069-015-0028-7 (PMC4551709; doi:10.1186/s13069-015-0028-7)
Supplement: Additional file 1: — Summary of human subject demographics and Table S1 legend. Table S1. Clinical features of SSc patients. Table S2. AA and Caucasian controls. [file 13069_2015_28_MOESM1_ESM.docx]

|  |
| --- |

**Methods for Supplemental Table 1**

Disease duration was determined based on when the first non-Raynaud phenomenon symptoms were documented. The following criteria were used to define visceral involvement: Pulmonary: Demonstration of abnormalities on high resolution computed tomographic scan (ground glass changes and/or fibrosis), pulmonary hypertension based on a right heart catheterization, restrictive changes on pulmonary function testing, or reduced diffusing capacity for carbon monoxide. GI: History of gastro-esophageal reflux disease based on either subjective and/or objective findings. Some patients had symptoms of reflux requiring treatment with a proton pump inhibitor and/or H2-antagonist. Others had abnormal motility documented by esophageal manometry or findings of esophagitis on upper endoscopy. Cardiac: Evidence on echocardiogram of left ventricular diastolic dysfunction, a pericardial effusion, elevated peak right ventricular systolic pressure, right ventricular and/or right atrial dilatation. Conduction abnormalities on a 12 – lead EKG was also considered sufficient. Renal: History of rapidly progressive renal failure. Antinuclear antibodies (ANA) and anti-centromere antibodies were determined by immunofluorescent analysis on Hep-2 cell substrates. Anti-Scl-70 (topoisomerase I) antibodies were determined by enzyme immunoassay.

| **Supplemental Table 2: AA and Caucasian Controls** | | | | |
| --- | --- | --- | --- | --- |
|  |  |  |  |  |
| Race | Gender | Donors | Smokers | Former Smokers |
| Caucasian | M | 14 | 1 | 2 |
| Caucasian | F | 23 | 1 | 2 |
| African-American | M | 6 | 0 | 1 |
| African-American | F | 28 | 1 | 1 |
|  |  |  |  |  |
| Age: Mean ± SD (range): | C | 38.8 ± 12.1 (18-64) | | |
|  | AA | 44.1 ± 12.8 (19-69) | | |
